# Structural and Energetic Determinants of Sweet Protein Recognition: Mechanistic Insights into Thaumatin Binding to the Human T1R2/T1R3 Receptor

**DOI:** 10.3390/ijms27094119

**Published:** 2026-05-05

**Authors:** Kikrusenuo Kiewhuo, Gulzaib Basharat, Thanyada Rungrotmongkol, Alisa Vangnai

**Affiliations:** 1 Center of Excellence in Biocatalyst and Sustainable Biotechnology, Department of Biochemistry, Faculty of Science, Chulalongkorn University, Bangkok 10330, Thailand; kikrutant@gmail.com; 2 Program in Bioinformatics and Computational Biology, College of Interdisciplinary and Integrative Studies, Chulalongkorn University, Bangkok 10330, Thailand; gullzaib79@gmail.com

**Keywords:** thaumatin, T1R2/T1R3 sweet taste receptor, GPCR, protein–protein recognition, structure–function relationship

## Abstract

Excessive sugar intake remains a major health challenge, motivating the development of safe and effective alternatives. Thaumatin, a natural high-intensity sweet protein, elicits sweetness through activation of the sweet taste receptor (T1R2/T1R3), yet its molecular recognition mechanism remains understudied. An integrated computational strategy combining comparative modeling, protein–protein docking, and 500 ns molecular dynamics simulations (triplicates) was employed to elucidate the thaumatin–receptor binding. Structural modeling identified the closed conformation of the Venus flytrap domain (VFT) as optimal for ligand engagement. Modeling revealed a stable binding interface characterized by electrostatic complementarity and van der Waals interactions, characterized by interfacial contacts of receptors and hydrogen bonding networks. Residue-level energy decomposition highlighted key residues (W418 and E422 of T1R2; S59 of T1R3) and thaumatin residues (K67, R82, and K137) that contribute substantially to complex stabilization, consistent with experimentally reported sweetness determinants. These findings provide molecular-level insight into sweet protein recognition and establish a structural framework for rational engineering of protein-based sweeteners with enhanced potency and selectivity.

## 1. Introduction

Excessive sugar intake is strongly linked to chronic lifestyle-related diseases, including cardiovascular disorders, obesity, type 2 diabetes, and non-alcoholic fatty liver disease, and it remains a major global public health concern [1,2]. This has driven intense efforts to develop low-calorie sugar substitutes with acceptable sweetness profiles. Current alternatives include synthetic sweeteners such as aspartame and sucralose, sugar alcohols, and naturally derived sweet proteins [3,4,5,6,7]. However, the rational design of effective sugar substitutes requires a detailed molecular understanding of how these compounds interact with the human sweet taste receptor (STR). The STR is a heterodimeric class C G protein-coupled receptor composed of T1R2 and T1R3 subunits. Each subunit consists of an extracellular Venus flytrap module (VFTM), a cysteine-rich domain (CRD), and a seven-transmembrane domain (TMD) [8,9,10]. Small-molecule sweeteners typically bind within the VFTM of T1R2 and T1R3. In contrast, the recognition mechanism of sweet-tasting proteins remains poorly understood, largely due to their larger size, complex surface features, and conformational flexibility [11].

The wedge model proposed by Temussi suggests that sweet proteins may not function as classical agonists but instead stabilize the active receptor conformation by interacting with a secondary binding site [12,13]. Despite the increasing number of alternative sweeteners reported over the past decades, only a limited number have achieved broad commercial adoption. This is often due to off-tastes, poor stability, or regulatory limitations. Among sweet proteins, thaumatin has attracted particular attention because of its exceptionally high sweetness potency, natural origin, and favorable safety profile [14]. Thaumatin is a 207-amino-acid protein with several high-resolution crystal structures available [15,16], making it an excellent model for structure–function studies. Previous mutagenesis experiments have identified several basic residues, most notably K67 and R82, as critical contributors to sweetness. Additional residues, including R76, K78, R79, and K137, also play important roles [17,18,19]. Substitution of these residues leads to pronounced reductions in sweetness, suggesting their involvement in receptor recognition. Notably, many of these residues cluster within a surface-exposed loop spanning residues 67–82, indicating the presence of a potential “sweetness hotspot” on the thaumatin surface.

In this study, we used an integrated in silico strategy to investigate the molecular interaction between thaumatin and the human T1R2/T1R3 sweet taste receptor. By combining molecular docking, AlphaFold-predicted receptor structures, and molecular dynamics (MD) simulations, we identified a previously unreported binding interface that directly involves key sweetness-determining residues of thaumatin. Comparison with earlier proposed binding models reveals a distinct interaction mode, supporting an alternative mechanism for receptor activation. These findings provide new molecular insights into sweet protein recognition and offer a structural basis for the rational design of next-generation sweeteners with improved sensory and functional properties.

## 2. Results

### 2.1. Quality Assessment and Conformational Selection of the Sweet Taste Receptor Model

**Figure 1:**
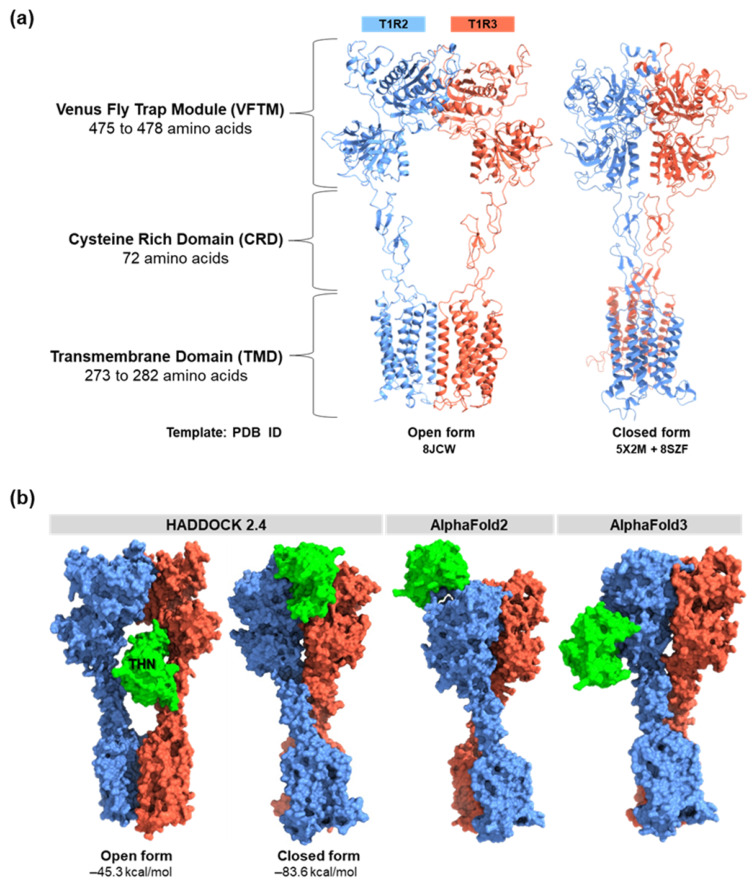
(**a**) Predicted 3D structures of the human T1R2/T1R3 heterodimer in open and closed conformations. T1R2 (blue) and T1R3 (red) domains (VFTM, CRD, and TMD) are indicated. The open model was built using 8JCW and the closed model using 5X2M and 8SZF. (**b**) Predicted binding modes of thaumatin (green) with T1R2 (blue) and T1R3 (orange). Docking results from HADDOCK 2.4 are shown on the left and complex predictions from AlphaFold2 Multimer and AlphaFold3 on the right.

Three-dimensional models of the T1R2/T1R3 sweet taste receptor heterodimer were constructed in both open and closed conformations using SWISS-MODEL. The closed-form model showed a sequence identity of 37.12% with the selected template structures, whereas the open-form model shared 29.38% identity (Figure 1a). Structural validation was performed using Ramachandran plot analysis and ERRAT quality assessment. The closed conformation demonstrated improved stereochemical properties, with 88.0% of residues located in the most favored regions and 99.3% within allowed regions. Only 0.7% of residues were found in disallowed regions. The open conformation showed 84.0% of residues in favored regions and 99.6% in allowed regions, with 0.4% in disallowed regions (Figure S1). ERRAT analysis yielded overall quality factors of 83.7% for the closed form and 83.3% for the open form, indicating comparable overall structural reliability.

Importantly, residues located in disallowed regions did not correspond to predicted functional or ligand-interacting residues, minimizing their potential impact on downstream analyses. The hybrid closed-form model was therefore selected based on its higher sequence identity, improved stereochemical quality, and superior domain-wise structural alignment relative to single-template models. Although regions outside the predicted binding interface may exhibit lower structural accuracy due to limited sequence identity, they are unlikely to significantly influence docking and MD simulations, as they do not directly participate in ligand recognition.

To further evaluate the plausibility of the predicted binding mode, comparisons were made with AlphaFold-derived complex structures. While alternative binding modes suggested by AlphaFold2-Multimer and AlphaFold3 cannot be excluded, the HADDOCK-derived model was prioritized based on (i) simultaneous engagement of both T1R2 and T1R3 subunits, (ii) consistency with experimentally validated interaction residues, and (iii) favorable interfacial energetics and hydrogen bonding patterns. These criteria provide an independent and mechanistically relevant basis for model selection beyond reliance on the wedge model hypothesis.

Taken together, the closed conformation was selected for subsequent protein–protein docking and MD simulations, as it represents the most structurally reliable and biologically plausible model. This selection is further supported by the proposed mechanism in which sweet proteins stabilize the receptor in a closed or near-closed conformation to promote activation.

### 2.2. Molecular Docking Reveals Preferential Thaumatin Binding to the Closed Receptor Conformation

To clarify the molecular basis of sweet protein recognition, protein–protein docking was performed using HADDOCK 2.4. Docking simulations were conducted for both the open and closed conformations of the T1R2/T1R3 receptor complex in interaction with thaumatin. Clear differences in binding orientation and interaction strength were observed between the two receptor states. The closed conformation yielded a substantially more favorable HADDOCK score (−83.60 kcal/mol) compared to the open conformation (−45.30 kcal/mol) (Figure 1b), indicating stronger and more stable intermolecular interactions in the closed state. Interface analysis revealed distinct binding regions for the two conformations. In the closed form, thaumatin primarily engaged the Venus flytrap module (VFTM) of T1R2. In contrast, docking to the open form is localized mainly to the cysteine-rich domain (CRD). The preferential interaction with the VFTM in the closed state aligns with the wedge model of receptor activation, which proposes that sweet proteins stabilize a closed, clamshell-like receptor conformation to trigger signaling [20].

The stronger docking score and favorable binding geometry in the closed state therefore support its functional relevance in sweetness perception. Based on these results, the closed thaumatin–receptor complex was selected for subsequent molecular dynamics simulations and energetic analyses. Key interfacial residues of the closed complex were identified using interaction mapping generated by LigPlot+ [21] (Figure S2). On the T1R2 subunit, residues D119, N120, L158, T409, C410, T411, V414, Y416, W418, Q419, and E422 contributed to the interaction interface. On T1R3, residues E48, P60, and D249 were involved. Thaumatin residues participating in binding included Y57, G64, I65, K67, D70, G72, G81, R82, G132, Q133, K137, P141, and G142.

### 2.3. Comparative Structural Evaluation Supports the HADDOCK-Derived Closed Complex

To assess the robustness of the predicted binding interface, a comparative analysis was performed using complex structures generated by AlphaFold2-Multimer, AlphaFold3, and HADDOCK 2.4. Full-length T1R2/T1R3 heterodimer models were predicted using both AlphaFold platforms and evaluated alongside the docking-derived open and closed conformations (Figure 1b). The AlphaFold2-Multimer model predicted a crossed heterodimer arrangement in which thaumatin binds exclusively to lobe 1 of the VFTM domain of T1R2, with no interaction observed with T1R3. In the AlphaFold3 model, thaumatin was positioned near lobe 2 of T1R2, again without significant engagement of T1R3. This asymmetric binding mode contrasts with the wedge model of receptor activation, which proposes that sweet proteins stabilize the receptor in a closed clamshell conformation through interactions involving both subunits [20,22]. Although AlphaFold-derived complexes exhibited high per-residue confidence (pLDDT) scores (Figure S3), confidence metrics alone do not guarantee biologically relevant interfacial arrangements. The predicted AlphaFold interfaces did not fully recapitulate experimentally supported mechanistic features and were therefore not prioritized for dynamic refinement.

**Figure 2:**
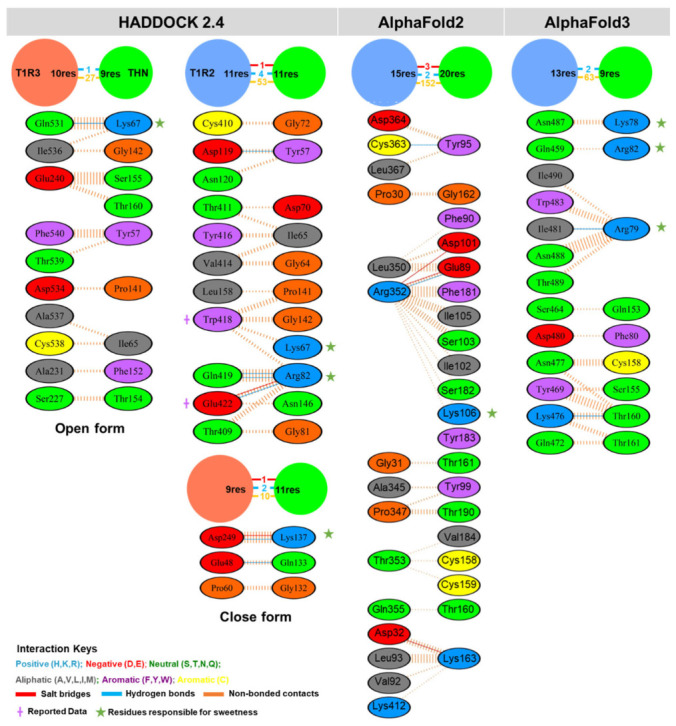
Residue-level interactions between thaumatin (THN) and the sweet taste receptor subunits T1R2 and T1R3 in complexes predicted by HADDOCK 2.4 (open and closed forms), AlphaFold2 Multimer, and AlphaFold3. Each interacting residue is color-coded by physicochemical property: positively charged (blue), negatively charged (red), neutral (green), aliphatic (gray), aromatic (purple), and cysteine (yellow). Interaction types are represented as follows: salt bridges (red lines), hydrogen bonds (blue lines), and non-bonded contacts (orange dashed lines). Previously reported receptor residues were marked with †, and experimentally key sweetness-related THN residues with a green star.

Interface mapping was performed for all four complexes using the PDBsum platform, and results are summarized in Figure 2 and Table S1. Among the models evaluated, only the closed-form complex predicted by HADDOCK exhibited simultaneous interaction with both T1R2 and T1R3, consistent with experimental evidence for cooperative receptor activation. This model also displayed the highest number of stabilizing interactions, including multiple hydrogen bonds and salt bridges. Key hydrogen bonds in the closed complex included D119–Y57, Q419–R82, and E422–R82 (T1R2–thaumatin), as well as E48–Q133 and D249–K137 (T1R3–thaumatin). Importantly, residues W418 and E422 of T1R2, previously reported as critical for sweet protein recognition [14], were involved only in the closed-form model. While all four models engaged at least one sweetness-associated residue on thaumatin, the closed-form complex captured a broader set of experimentally validated residues, including K67, R82, and K137. Taken together, the closed thaumatin–T1R2/T1R3 complex predicted by HADDOCK 2.4 best satisfied both structural plausibility and biological relevance. It uniquely reproduced dual-subunit engagement, incorporated key experimentally supported residues, and demonstrated the most extensive stabilizing interactions. Accordingly, this model was selected for subsequent molecular dynamics simulations and binding free energy analyses.

### 2.4. MD Simulations Confirm Stability of the Closed Thaumatin–Receptor Complex

**Figure 3:**
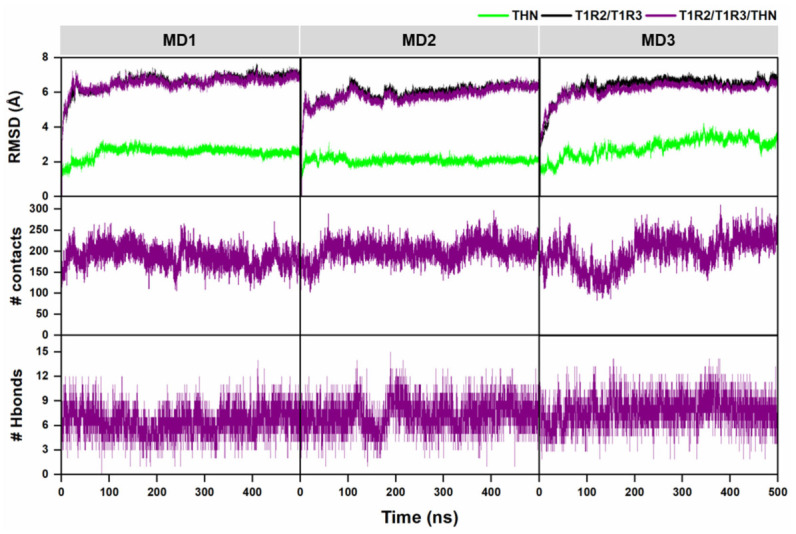
(**Top**) RMSD plots of the receptor (T1R2/T1R3, black), thaumatin (THN, green), and the overall complex (magenta) over 500 ns performed in triplicate. (**Middle**) Time evolution of atomic contacts between THN and the receptor complex over 500 ns (triplicates). (**Bottom**) Intermolecular hydrogen bonds between THN and the receptor complex over 500 ns (triplicates).

To evaluate the dynamic stability and conformational behavior of the closed-form thaumatin–T1R2/T1R3 complex, 500 ns molecular dynamics simulations were performed in triplicate. Structural stability was first assessed using root mean square deviation (RMSD) analysis. As shown in Figure 3, all three replicas exhibited an initial increase in RMSD during the equilibration phase, followed by convergence and stabilization after approximately 200 ns. The T1R2/T1R3 receptor displayed RMSD values ranging from 5.9 to 7.1 Å, reflecting expected flexibility within the Venus flytrap module. In contrast, thaumatin maintained lower RMSD values between 2.0 and 3.3 Å, indicating preserved structural integrity and a stable binding orientation throughout the simulations. Interfacial stability was further examined by monitoring atomic contacts between thaumatin and the receptor. Although early trajectory segments showed dynamic rearrangements, the average number of contacts remained above 200 across all replicas, supporting persistent intermolecular engagement. The number of intermolecular hydrogen bonds was also evaluated as an indicator of interaction specificity and complex stabilization. Approximately six hydrogen bonds were consistently maintained throughout the simulations, reinforcing the stability of the binding interface. The radius of gyration (Rg) was calculated to assess global compactness. The receptor maintained a stable Rg between 28 and 30 Å, while thaumatin exhibited a consistent Rg of 16–17 Å across all runs (Figure S4). These values indicate that neither protein underwent major structural unfolding nor destabilization during the simulation.

### 2.5. Residue-Level Energetic Decomposition and Interaction Network Analyses Define Key Sweetness Determinants

To further validate the stability and biological relevance of the thaumatin–T1R2/T1R3 complex, binding free energy calculations were performed using the solvated interaction energy (SIE) method based on the final 100 ns of each MD trajectory (Table 1). Across three independent simulations, the predicted binding free energies were consistent, ranging from −14.26 to −15.51 kcal/mol, indicating reproducible and moderately strong protein–protein interactions. The Coulombic term E_c_(D_in_) and the reaction field contribution ΔG^R^ are both large in magnitude but opposite in sign, with E_c_(D_in_) ranging from −430 to −535 kcal/mol and ΔG^R^ from +460 to +540 kcal/mol across the three simulations. This substantial compensation results in a relatively small net electrostatic contribution to the overall binding free energy. In contrast, van der Waals interactions ΔE_vdW_ (−102 to −119 kcal/mol) and nonpolar solvation contributions γ⋅ΔMSA(ρ) (−20 to −23 kcal/mol) consistently provide favorable stabilization. These terms are not significantly offset and therefore represent the primary contributors to the net binding affinity. Together, these results indicate that although electrostatic interactions are strong at the interface, they are largely balanced by solvent screening effects. Consequently, the overall binding free energy is governed by the combined contribution of van der Waals interactions and nonpolar solvation, with electrostatics playing a key but compensatory role.

**Figure 4:**
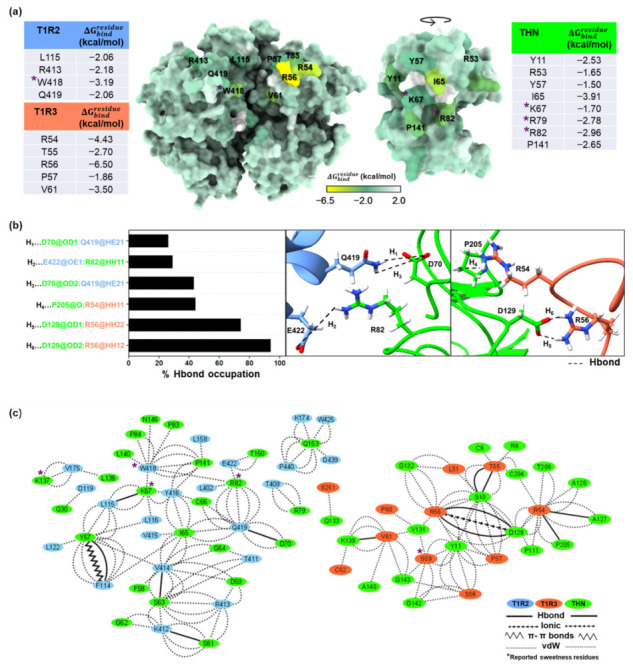
(**a**) Per-residue free energy decomposition (${\Delta G}_{bind}^{residue}$) of the thaumatin–T1R2/T1R3 complex. Receptor residues with ${\Delta G}_{bind}^{residue}$ < −1.5 kcal/mol are mapped onto the complex surface and listed in the corresponding tables. The color gradient (yellow to teal) reflects decreasing energetic contribution from strong to weak interactions. (**b**) Representative intermolecular hydrogen bonds and their occupancy. The bar plot (**left**) shows the percentage occupancy of key interfacial residue pairs, while the central and right panels illustrate the spatial arrangement of the most prominent hydrogen bond interactions. (**c**) Residue-level intermolecular interaction network between T1R2, T1R3, and thaumatin (THN), highlighting dynamic contacts formed during simulation.

For detailed interface characterization, per-residue free energy decomposition was performed using MM/GBSA. The average binding energy contributions for the first simulation are presented in Figure 4a, while those for the second and third runs are provided in Figures S5. Stabilizing residues (${\Delta G}_{bind}^{residue}$ < −1.5 kcal/mol) were predominantly located at the predicted binding interface. In T1R2, major contributors included L115, N120, L122, L158, V175, T409, C410, T411, K412, R413, V414, Y416, W418, Q419, and E422. In T1R3, significant residues comprised L51, R54, T55, R56, P57, S59, V61, K111, and V366. On the thaumatin side, strong contributors included R8, Y11, R29, R53, Y57, I65, K67, L74, K78, R79, R82, K106, R125, D129, V131, A136, K137, K139, P141, F152, Q153, K163, R171, and R175.

Hydrogen bond analysis further emphasized the role of electrostatic complementarity in complex stabilization. In T1R2, Q419 and E422 formed stable hydrogen bonds with D70 and R82 of thaumatin, exhibiting occupancies of 26–43% and distances of 2.78–2.86 Å (Figure 4b). In T1R3, R54 and R56 formed prominent hydrogen bonds with D129 and P205 of thaumatin, with occupancies reaching up to 94%. Extended analyses of the additional replicas confirmed the persistence of these interactions and revealed additional stabilizing contacts involving D119 and T411 (T1R2) and D129 (thaumatin) (Figure S6). Notably, approximately 22% of the hydrogen bonds involved arginine residues, highlighting the dominant role of positively charged side chains in mediating receptor engagement [23]. To provide a systems-level view of the intermolecular contacts, residue interaction networks were generated using the RING server and visualized with Cytoscape (Figure 4c). In this representation, residues were treated as nodes and interactions as edges, enabling the identification of conserved and dynamic contacts across simulations. Network analysis confirmed the same key interactions observed in MM/GBSA and hydrogen bond analyses, particularly involving W418 (T1R2) and S59 (T1R3) with K67, R82, and K137 of thaumatin through hydrogen bonding, van der Waals, and π–cation interactions (Figure S7). The complete inter- and intramolecular interaction patterns across all three runs are summarized in Table S2.

### 2.6. Collective Dynamics and Structural Rigidity at the Binding Interface

**Figure 5:**
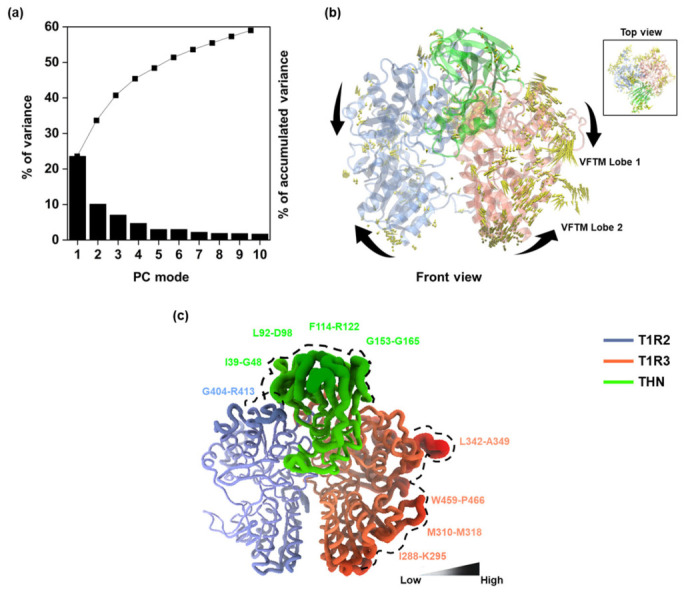
(**a**) The PCA plot; (**b**) front and top (inset) views show porcupine plots of quantitative characters of MD1. (**c**) B-factor representation of the T1R2/T1R3–THN complex; structure is ranked from rigid (lighter gradient) to flexible (darker gradient). The residues with high fluctuations corresponding to high B-factor values have been highlighted.

Principal component analysis (PCA) was performed to characterize the dominant collective motions of the thaumatin–T1R2/T1R3 complex using 100 snapshots extracted from the final 100 ns of each MD trajectory. The scree plot for MD run 1 is shown in Figure 5a, while those for runs 2 and 3 are provided in Figure S8. Across all three simulations, the first principal component (PC1) was dominant among the first ten modes, accounting for 23.57%, 12.14%, and 25.68% of the total variance in runs 1, 2, and 3, respectively. The accumulated variance indicates that the motion of the complex is primarily governed by one or two large-scale collective movements rather than random fluctuations. Runs 1 and 3 exhibited higher variance contributions from PC1, whereas run 2 showed a more uniform distribution of variance across multiple modes. This behavior is consistent with the higher intrachain interaction density observed in run 2, which may restrict large-amplitude motions and distribute conformational variability across several modes. Projection of the dominant motion onto the structure (Figure 5b) revealed that T1R3 adopts a comparatively more compact conformation than T1R2. The two lobes of the Venus flytrap module (VFTM) in both receptor subunits were oriented toward each other, representing a closed clamshell-like architecture consistent with an active binding configuration.

Structural flexibility was further examined through temperature factor (B-factor) analysis derived from the same 100-frame ensemble (Figure 5c). The B-factor, reflecting residue-level root mean square fluctuation (RMSF), provides insight into local mobility and conformational adaptability [24]. Regions with elevated B-factor values were predominantly located in solvent-exposed loops, including residues G404–R413 in T1R2; I288–K295, M310–M318, L342–A349, and W459–P466 in T1R3; and I39–G48, L92–D98, F114–R122, and G153–G165 in thaumatin. Importantly, these highly flexible segments did not directly participate in the protein–protein interface, apart from R413 in T1R2. This observation suggests that interfacial regions remain relatively rigid, thereby preserving key contact points essential for complex stability. Similar flexibility patterns were observed across independent trajectories (Figure S9), further confirming the reproducibility of dynamic behavior. Overall, PCA and B-factor analyses demonstrate that the thaumatin–receptor complex is characterized by coordinated large-scale motions and reduced interfacial flexibility. T1R2 exhibited lower flexibility at the binding interface compared to T1R3, consistent with its stronger energetic contributions observed in the binding free energy analysis.

## 3. Discussion

The closed thaumatin–T1R2/T1R3 complex selected for the molecular dynamics simulations and binding free energy analyses from comparative structural evaluation displayed the interaction of several previously reported sweetness-determining residues—particularly K67, R82, and K137 of thaumatin, as well as W418 of T1R2, which were directly observed to be engaged at the interface [14,19,25]. The involvement of multiple positively charged residues (lysine and arginine) is especially significant, as these residues have been shown experimentally to be critical for sweetness induction. Their direct participation in receptor binding supports the functional relevance of the predicted interface and provides structural evidence for the role of electrostatic interactions in sweet protein recognition.

The MD simulations analysis, including the RMSD, contact analysis, hydrogen bond persistence, and Rg profiles, demonstrates that the thaumatin–sweet taste receptor complex remains structurally stable over extended simulation times. The preservation of key interfacial interactions further supports the biological plausibility of the closed-state binding model. The energetic decomposition of the key sweetness determinants, including several experimentally validated sweetness-determining residues K67, K78, R79, R82, and K137 of thaumatin, together with W418 and E422 of T1R2, showed substantial stabilizing contributions. Previous mutagenesis studies have demonstrated that substitution of these residues markedly reduces sweetness intensity [16,17,19,25]. Their energetic importance in this study provides strong structural support for their functional role in receptor recognition and activation. In contrast, acidic residues such as D55 and D60 showed weak or unfavorable contributions, consistent with reports indicating that their mutation has minimal impact on sweetness [15]. These residues are well-established contributors to sweetness induction, and their reproducible engagement across all simulations reinforces the biological plausibility of the predicted interface. Overall, the convergence of binding free energy calculations, residue-level energetic decomposition, hydrogen bonding persistence, and interaction network analysis demonstrates that the interaction is driven by a balance of electrostatic complementarity and van der Waals/nonpolar stabilization between thaumatin and both receptor subunits.

The consistent involvement of experimentally validated sweetness-determining residues provides strong mechanistic support for the proposed binding model and its relevance to sweet taste receptor activation. Thaumatin also exhibited coordinated movement with the receptor throughout the trajectory, suggesting strong dynamic coupling between ligand and receptor subunits. This correlated motion supports the hypothesis that sweetness induction involves stabilization of a specific conformational ensemble rather than transient binding events. The PCA and B-factor findings reinforce the structural stability of the proposed binding model and suggest that conformational stabilization of the receptor may be a critical prerequisite for effective sweetness signaling [26,27].

Although this study presents a detailed computational investigation of thaumatin binding to the human sweet taste receptor (T1R2/T1R3), several limitations should be acknowledged. Firstly, the model relies on a homology-derived structure of the T1R2/T1R3 receptor, which, despite being built upon a robust template, may not fully capture the detailed dynamics of the native protein. Secondly, the accuracy of the binding site is not validated using experimental data. Thus, further experimental validation, such as site-directed mutagenesis studies or crystallographic analyses of the thaumatin–receptor complex, would significantly strengthen the proposed binding model. Findings of this study provide important insights into the molecular basis of sweetness perception and a foundation for the rational design of novel sweeteners. While the present study focuses on sweet protein recognition, it also provides implications for small-molecule ligand binding. The open form may represent the initial ligand-accessible state, while the closed form corresponds to the signaling-competent conformation. Therefore, consideration of both states could provide a more comprehensive framework for virtual screening and structure-based design.

## 4. Materials and Methods

### 4.1. Structural Modeling and Preparation of the T1R2/T1R3 Sweet Taste Receptor

Three-dimensional models of the human sweet taste receptor heterodimer (T1R2/T1R3) were generated using SWISS-MODEL [28]. The amino acid sequences of human T1R2 and T1R3 were retrieved from the UniProt Knowledgebase (UniProtKB IDs: Q8TE23 and Q7RTX0, respectively). Template selection was guided by sequence identity, structural coverage, and functional relevance, with particular emphasis on the extracellular Venus flytrap module (VFTM) and cysteine-rich domain (CRD), which are critical for ligand recognition. To capture receptor conformational variability, both closed and open states of the T1R2/T1R3 complex were constructed. The closed conformation was modeled using a hybrid template strategy that combined the VFTM ligand-binding domains of the medaka fish T1R2a–T1R3 receptor (PDB ID: 5X2M) and the CRD + TMD of the active-state human calcium-sensing receptor (CaSR) bound to cinacalcet in lipid nanodiscs (8SZF) [29,30]. The open conformation was derived from the AlphaFold2-predicted structure of the full-length human T1R2/T1R3 receptor (PDB ID: 8JCW) [31]. Model quality was assessed using Ramachandran plot analysis and the ERRAT quality factor via the SAVES v6.0 server [32,33]. The crystal structure of thaumatin, a 207-amino-acid sweet protein, was retrieved from the Protein Data Bank (PDB ID: 1THV) [34]. This structure was used as the ligand model in subsequent protein–protein docking and interaction analyses. Protonation states of ionizable residues were assigned at physiological pH (7.4) using PROPKA 3.0 to ensure accurate electrostatic representation for subsequent docking and molecular dynamics simulations [35].

### 4.2. Protein–Protein Docking and Model Selection

Prior to docking, structural preprocessing of the T1R2/T1R3 receptor models and thaumatin (1THV) was carried out using UCSF ChimeraX, University of California, San Francisco, CA, USA [36] and OpenBabel [37]. Preparation steps included the removal of crystallographic water molecules, the addition of hydrogen atoms, the correction of structural inconsistencies where necessary, and file format conversion for compatibility with the docking platform. The binding site for the protein complex was selected based on the sweetness-inducing and interacting residues reported in previous literature. Protein–protein docking was performed using HADDOCK 2.4, an information-driven docking approach that allows flexible modeling of biomolecular complexes [38,39]. The docking workflow consisted of three sequential stages: (i) rigid-body energy minimization (it0), (ii) semi-flexible refinement (it1), and (iii) final refinement in explicit solvent. During the rigid-body stage, multiple orientations of thaumatin relative to the receptor were generated to sample potential binding configurations. The best-ranked poses were subsequently refined through torsion-angle molecular dynamics, enabling flexibility in backbone and side-chain atoms at the interaction interface. The final refinement step involved short molecular dynamics simulations in an explicit water environment, followed by energy minimization to account for solvation effects. Docked complexes were clustered based on interface root mean square deviation (iRMSD), clustering cutoff 7.5 Å, and ranked using the HADDOCK scoring function; see Equation (1):

HADDOCK score = 1.0 × E_vdw_ + 0.2 × E_ele_ + 1.0 × E_desol_ + 0.1 × E_air_ (1)

where E_vdw_ represents intermolecular van der Waals energy, E_ele_ is electrostatic energy, E_desol_ denotes empirical desolvation energy, and E_air_ corresponds to the restraint violation energy derived from ambiguous and unambiguous interaction restraints. The final docking models were evaluated based on HADDOCK score, cluster size, and interface interaction patterns to identify the most plausible binding mode of thaumatin with the sweet taste receptor.

### 4.3. AlphaFold-Based Complex Structure Prediction and Interface Analysis

The structure of the thaumatin–sweet taste receptor complex was predicted using both AlphaFold2-Multimer [40] and AlphaFold3 [41], which enable high-confidence modeling of multi-chain protein assemblies. AlphaFold2-Multimer, an extension of AlphaFold2, is optimized for predicting protein–protein complexes, including heterodimers and higher-order assemblies. AlphaFold3 further expands these capabilities by incorporating non-protein components such as nucleic acids and small molecules while improving the modeling of multi-component interactions. For AlphaFold2-Multimer predictions, models were generated through the Cosmic^2^ science gateway [42], using default multiple sequence alignment (MSA) settings and the full sequence database option (--full_dbs). The top-ranked model, selected based on the ranking confidence score, was used for subsequent analyses. Model reliability was assessed using AlphaFold confidence metrics, including the predicted TM-score (pTM), which reflects global structural accuracy, and the inter-chain predicted TM-score (ipTM), which evaluates interfacial packing between subunits. Additional complex structures were obtained using the AlphaFold3 web server, which generates five structural predictions per submission. The full-length sequences of T1R2 (UniProtKB: Q8TE23), T1R3 (Q7RTX0), and thaumatin were used as inputs for both AlphaFold2-Multimer and AlphaFold3 predictions. Per-residue structural confidence was evaluated using predicted local distance difference test (pLDDT) scores to assess local model reliability. To characterize the intermolecular interface and identify interacting residues within the predicted complexes, interaction analysis was performed using the PDBsum server [43]. This platform provides detailed summaries of interface contacts, hydrogen bonds, salt bridges, and secondary structure features, enabling systematic evaluation of protein–protein interactions.

### 4.4. Molecular Dynamics Simulations and Binding Free Energy Analysis

All molecular dynamics (MD) simulations were performed using the AMBER 22 software suite, University of California, San Francisco, CA, USA [44,45]. Protein components were parameterized with the ff19SB force field [46]. Note that to reduce computational cost while preserving the key ligand-recognition region, only the Venus flytrap module (VFTM) of the sweet taste receptor was included in the simulations in complex with thaumatin. The solvated system underwent a two-stage energy minimization procedure: 3000 steps of steepest descent minimization followed by 1500 steps using the conjugate gradient algorithm. A 10 Å cutoff was applied for non-bonded interactions. Bonds involving hydrogen atoms were constrained using the SHAKE algorithm [47], and long-range electrostatic interactions were treated using the Particle Mesh Ewald (PME) method [48]. The system gradually heated to 298 K over 100 ps under the canonical (NVT) ensemble, followed by 200 ps of equilibration at 298 K to ensure structural stability. Production simulations were then carried out for 500 ns under isothermal–isobaric (NPT) conditions. Trajectory analyses were conducted using the CPPTRAJ module in AMBER. Structural stability and dynamic behavior were evaluated through root mean square deviation (RMSD), radius of gyration (Rg), intermolecular hydrogen bond analysis, and inter-residue contact calculations. These metrics provided insight into the conformational stability and binding persistence of the thaumatin–receptor complex throughout the simulation. Binding free energy (ΔGbind) was estimated over the final 100 ns of the trajectory using the solvated interaction energy (SIE) method [49], accounting for van der Waals, electrostatic, and solvation (polar and nonpolar) contributions. Per-residue free energy decomposition was performed with MMPBSA.py [50] to identify key residues contributing to complex stabilization at the protein–protein interface based on classical molecular mechanics and continuum solvation models. Principal component analysis (PCA) was conducted using the same MD trajectories to characterize dominant collective motions. Residue interaction networks were generated using the RING web server (accessed 24 May 2024) [51] with default parameters, including a hydrogen bond cutoff of 3.5 Å, salt bridge cutoff of 4.0 Å, van der Waals cutoff of 0.5 Å, π–π stacking cutoff of 6.5 Å, π–cation cutoff of 5.0 Å, and disulfide bond cutoff of 2.5 Å. The resulting interaction networks were visualized using Cytoscape 3.10.4, San Diego, CA, USA [52].

## 5. Conclusions

This study elucidates the molecular basis of thaumatin recognition by the human sweet taste receptor (T1R2/T1R3) through integrated structural modeling, docking, molecular dynamics simulations, and energetic analyses. Comparative modeling supported the closed conformation of the receptor as the most structurally reliable and functionally relevant state. Docking revealed a pronounced preference for thaumatin binding within the Venus flytrap module (VFTM) of the closed receptor, consistent with the wedge model of activation. In contrast, AlphaFold-based complex predictions did not reproduce the experimentally supported dual-subunit engagement observed in the HADDOCK-derived closed complex. Triplicate 500 ns molecular dynamics simulations confirmed the stability of the closed thaumatin–receptor complex, demonstrating persistent intermolecular contacts and hydrogen bonding. Binding free energy calculations yielded consistent affinities (−14.26 to −15.51 kcal/mol). Electrostatic interactions were strong but largely compensated by solvent screening, while van der Waals and nonpolar interactions provided the primary net stabilization. Residue-level decomposition identified key contributors at the interface, including W418 and E422 of T1R2, S59 of T1R3, and K67, R82, and K137 of thaumatin—residues previously validated as critical for sweetness induction. Interaction network analysis further underscored the central role of positively charged residues in mediating receptor engagement. Principal component and flexibility analyses revealed coordinated collective motions and reduced interfacial mobility, indicating that thaumatin binding stabilizes a closed clamshell-like conformation of the receptor. Together, these findings establish a coherent structural and energetic framework for sweet protein recognition and provide mechanistic insight to guide the rational engineering of next-generation protein-based sweeteners with enhanced potency and selectivity.

## Figures and Tables

**Table 1:** Solvated interaction energy (SIE) components and calculated binding free energies for the thaumatin–T1R2/T1R3 complex estimated from the last 100 ns of the MD trajectories.

	MD1	MD2	MD3
E_c_(D_in_)	−482.68 ± 71.44	−534.53 ± 158.59	−430.97 ± 88.48
ΔG^R^	500.49 ± 74.70	542.77 ± 160.14	463.99 ± 104.20
ΔE_vdW_	−116.35 ± 32.77	−102.44 ± 44.70	−118.93 ± 39.10
γ⋅ΔMSA(ρ)	−21.93 ± 6.03	−19.98 ± 8.74	−22.63 ± 7.20
Constant (C)	−2.89		
* ∆G_bind_	−15.51 ± 3.60	−14.85 ± 5.40	−14.26 ± 3.03

* ΔG_bind_ = α[E_c_(D_in_) + ΔG^R^ + ΔE_vdW_ + γ⋅ΔMSA(ρ)] + C, where $E_{c}\left(D_{in}\right)$ is the Coulombic interaction energy calculated with an interior dielectric constant $D_{in}$, $\Delta G^{R}$ is the reaction field (polar solvation) contribution, $\Delta E_{\mathrm{vdW}}$ is the van der Waals energy, and γ⋅ΔMSA(ρ) represents the nonpolar solvation term proportional to the change in molecular surface area. The parameters were set to α=0.1048, γ=0.012894 kcal·mol^−1^·Å^−2^, $D_{in}=2.25$, and C=−2.89 kcal·mol^−1^.

## Data Availability

The original contributions presented in this study are included in the article/Supplementary Material. Further inquiries can be directed to the corresponding authors.

## Supplementary Materials

The following supporting information can be downloaded at: https://www.mdpi.com/article/10.3390/ijms27094119/s1.

## Abbreviations

The following abbreviations are used in this manuscript:

STRs	Sweet Taste Receptors
VFTM	Venus Flytrap Module
CRD	Cysteine-Rich Domain
TMD	Transmembrane Domain
CaSR	Calcium-Sensing Receptor
Irmsd	Interface Root Mean Square Deviation
MSA	Multiple Sequence Alignment
pTM	TM-Score
ipTM	Inter-Chain Predicted TM-score
pLDDT	Predicted Local Distance Difference Test
MD	Molecular Dynamics Simulations
HADDOCK	High-Ambiguity-Driven Protein–Protein Docking
PME	Particle Mesh Ewald
RMSD	Root Mean Square Deviation
Rg	Radius of Gyration
SIE	Solvated Interaction Energy
PCA	Principal Component Analysis
THN	Thaumatin
MM/GBSA	Molecular Mechanics/Generalized Born Surface Area
RMSF	Root Mean Square Fluctuation
