# Supplementary material for: Structural and Energetic Determinants of Sweet Protein Recognition: Mechanistic Insights into Thaumatin Binding to the Human T1R2/T1R3 Receptor
Source: Int J Mol Sci. 2026 May 5;27(9):4119. doi: 10.3390/ijms27094119 (PMC13163733; doi:10.3390/ijms27094119)
Supplement: Supplementary file 1 [file ijms-27-04119-s001.zip › ijms-4258931-supplementary.pdf]

Electronic supplementary information

Article

Structural and Energetic Determinants of Sweet Protein Recognition: Mechanistic Insights into Thaumatin Binding to the Human T1R2/T1R3 Receptor

Kikrusenuo Kiewhuo [1], Gulzaib Basharat [2], Thanyada Rungrotmongkol [1,2*] and Alisa Vangnai [1*]

1 Center of Excellence in Biocatalyst and Sustainable Biotechnology, Department of Biochemistry, Faculty of Science, Chulalongkorn University, Bangkok, Thailand
2 Program in Bioinformatics and Computational Biology, College of Interdisciplinary and Integrative Studies, Chulalongkorn University, Bangkok, Thailand
* Correspondence: thanyada.r@chula.ac.th (TR) Tel.: ; alisa.v@chula.ac.th (AV) Tel.:

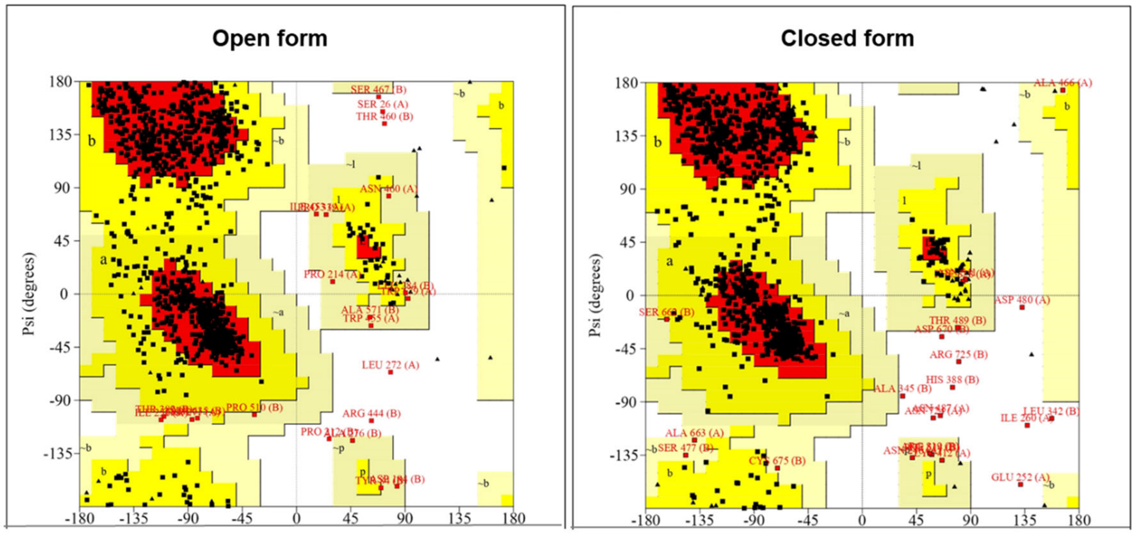

Residues in the most favored region = 84.0%
Residues in the additional allowed region = 14.6 %
Residues in the generously allowed region = 1.0%
Residues in the disallowed region = 0.4%

Residues in the most favored region = 88%
Residues in the additional allowed region = 10.5 %
Residues in the generously allowed region = 0.8%
Residues in the disallowed region = 0.7%

Figure S1. Ramachandran plots for the sweet taste receptor in its open-form model (left) and closed-form model (right) conformations generated using PROCHECK

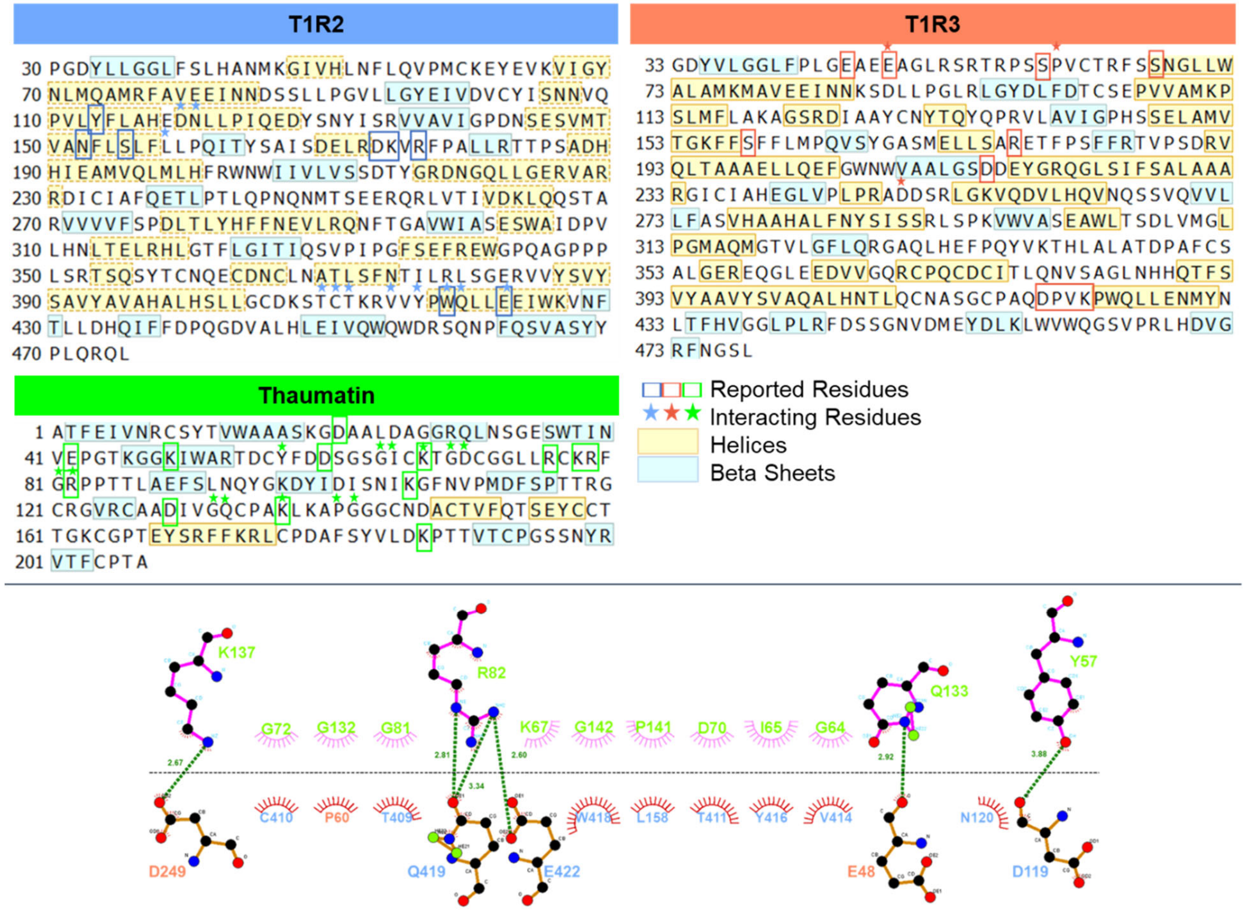

Figure S2. The interacting residues of T1R2/T1R3 and thaumatin reported in various studies have been highlighted along with the interacting residues obtained from docking in this study. Dimplot of protein-protein complex between STR (T1R2/T1R3) and thaumatin.

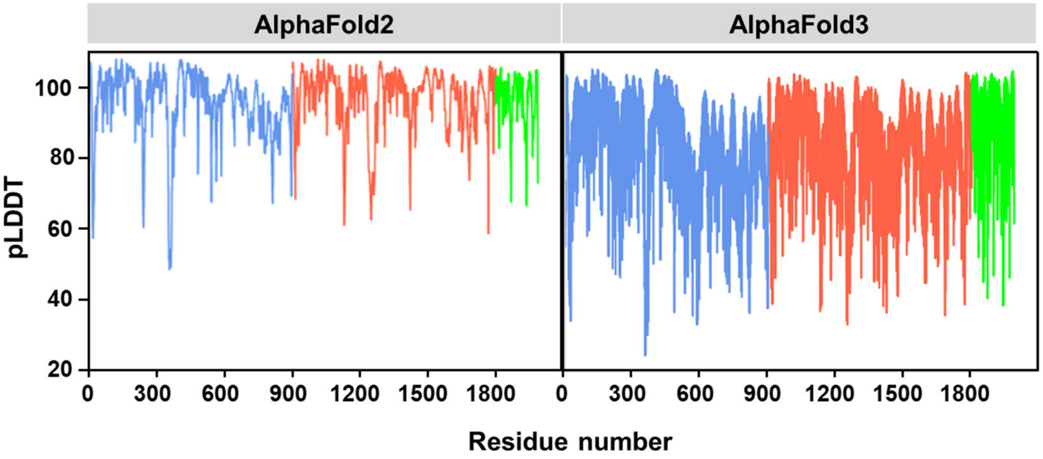

Figure S3. The predicted LDDT per residue for the top rank AlphaFold2 Multimer and AlphaFold3 model

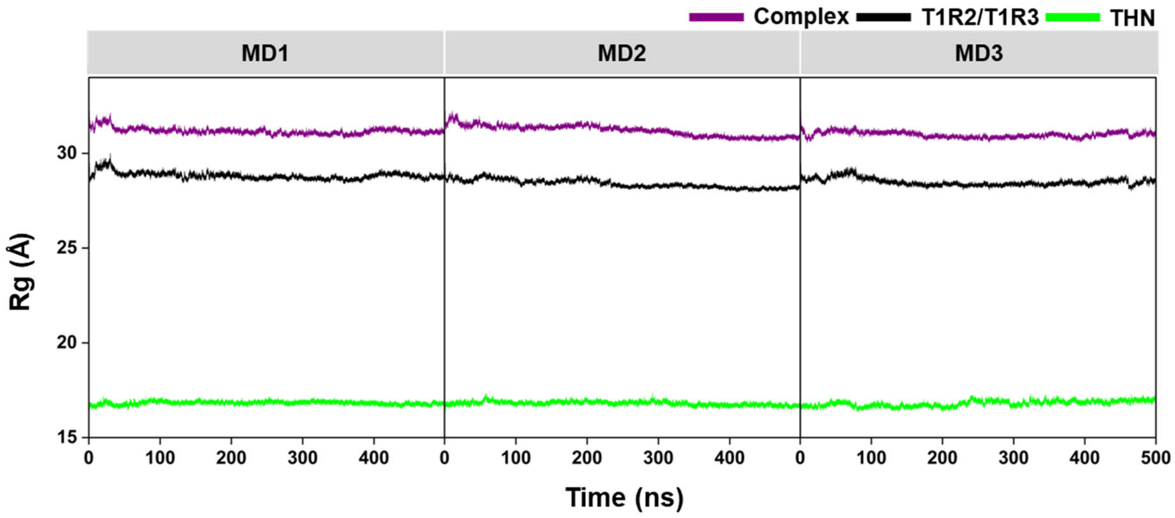

Figure S4. Radius of Gyration (Rg) of MD simulation for a timescale of 500 ns. In all systems, the color code indicates – complex (purple), T1R2/T1R3 (black), thaumatin (green).

(a)

T1R2	$\Delta G_{bind}^{residue}$ (kcal/mol)
V175	-2.61
T409	-1.86
K412	-2.65
*W418	-3.09
*E422	-3.19

T1R3	$\Delta G_{bind}^{residue}$ (kcal/mol)
L51	-2.08
R56	-6.08

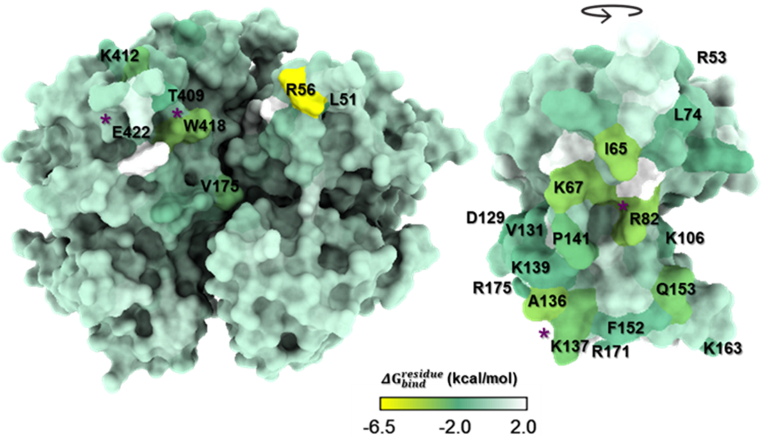

THN	$\Delta G_{bind}^{residue}$ (kcal/mol)
R53	-1.84
I65	-3.38
K67	-3.28
L74	-1.76
*R79	-1.70
*R82	-3.40
K106	-1.52
D129	-2.03
V131	-1.65
A136	-3.36
*K137	-2.74
K139	-1.76
P141	-2.41
F152	-1.60
Q153	-2.81
K163	-1.52
R171	-1.63
R175	-1.88

(b)

T1R2	$\Delta G_{bind}^{residue}$ (kcal/mol)
N120	-1.62
L122	-2.11
L158	-1.50
C410	-2.46
T411	-2.85
R413	-4.12
V414	-2.04
Y416	-2.36
*W418	-1.90

T1R3	$\Delta G_{bind}^{residue}$ (kcal/mol)
R54	-5.77
T55	-2.45
R56	-8.72
*S59	-1.65
K111	-2.25
V366	-1.89

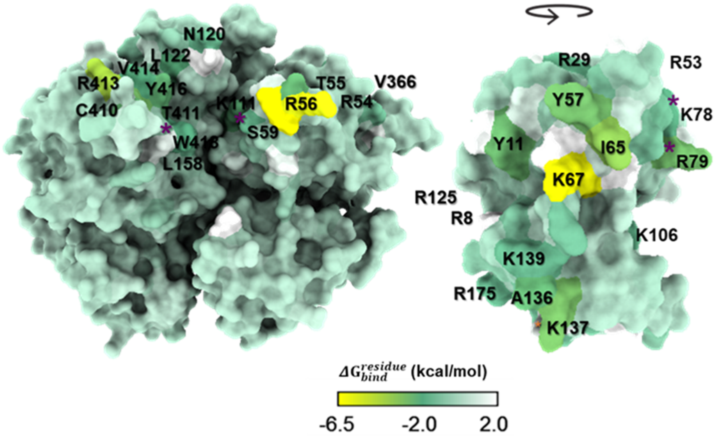

THN	$\Delta G_{bind}^{residue}$ (kcal/mol)
R8	-1.83
Y11	-2.59
R29	-1.66
R53	-2.89
Y57	-2.77
I65	-3.47
K67	-6.26
*K78	-1.83
*R79	-2.75
K106	-1.72
R125	-1.50
A136	-2.39
*K137	-2.89
K139	-1.63
R175	-1.64

Figure S5. (a) MD2 and (b) MD3 per residue decomposition free energy (kcal/mol) of the STR (T1R2/T1R3) and thaumatin (THN) calculated with the MMGBSA method from last 100 ns.

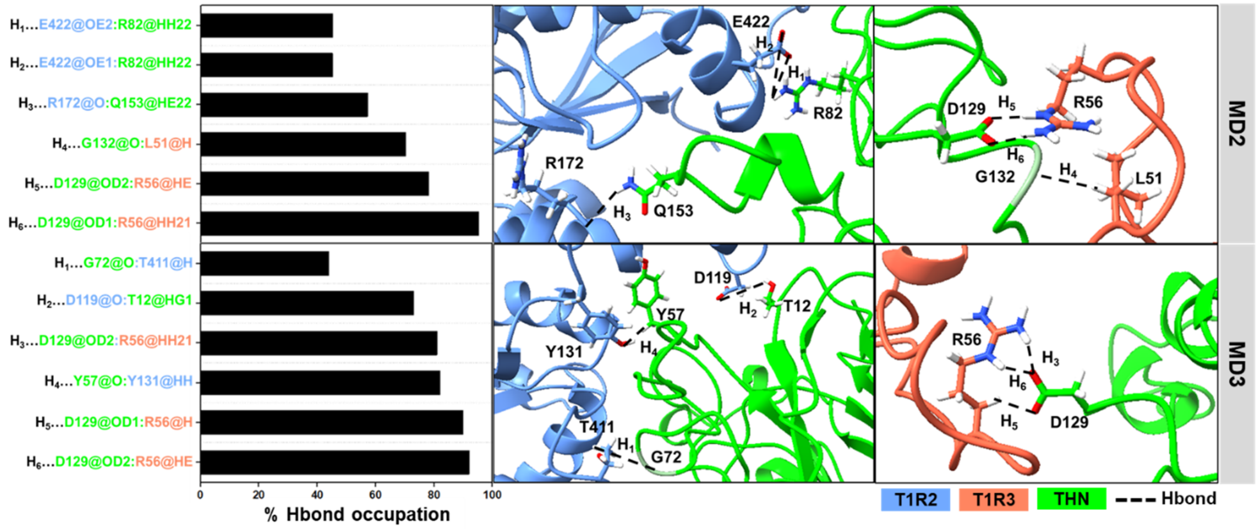

Figure S6. Percentage interaction occupation of the hydrogen bond interactions between the STR (T1R2/T1R3) and thaumatin (THN) from MD2-3. The figure on the right shows a close-up of thaumatin interacting with the STR. The H-bonds and distance (Å) have been represented in cyan dotted line.

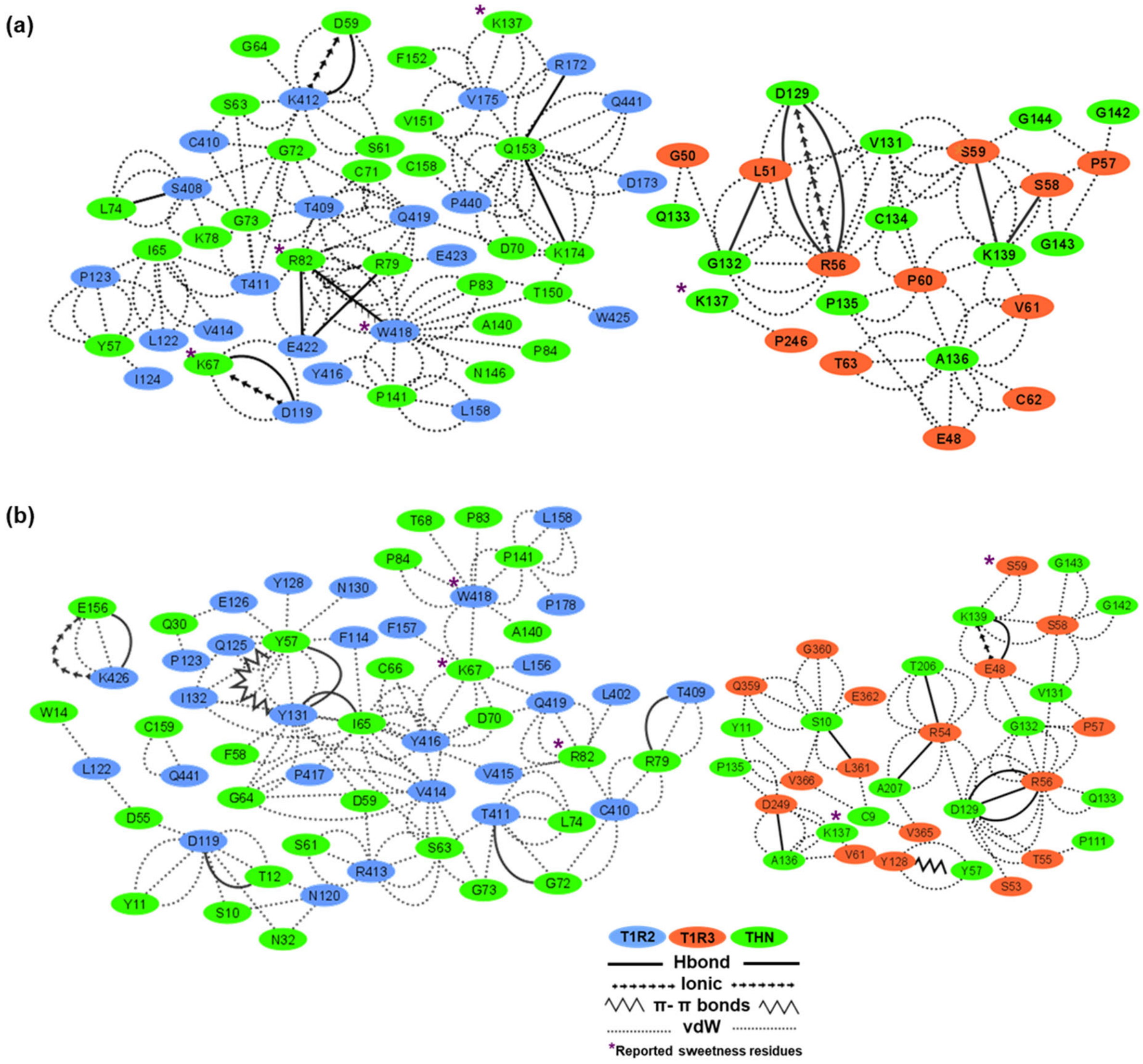

Figure S7. (a) MD2 and (b) MD3 residual intermolecular network showing dynamic residue interaction between T1R2 (blue), T1R3 (orange) and thaumatin (green). The interactions show the formation of vdW, hbonds, ionic and π- π bonds between the proteins. The previously reported sweetness inducing residues have been marked with asterisk (*).

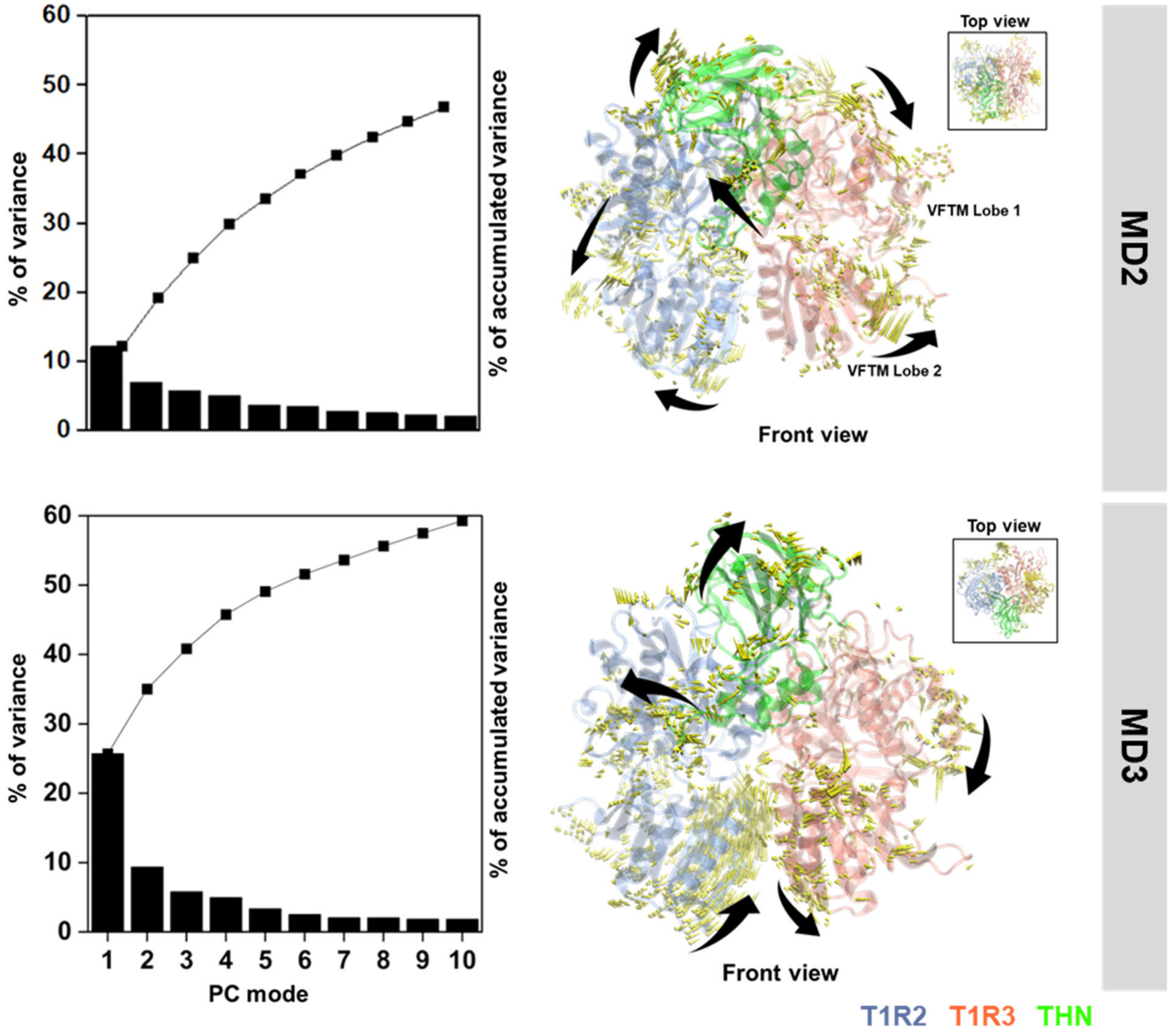

Figure S8. PCA screen plot and front and top view (inset) porcupine plot of quantitative characters of MD1. The porcupine plot demonstrates significant motion of the proteins (the arrowhead denotes direction of motion while length indicates the amplitude of motion).

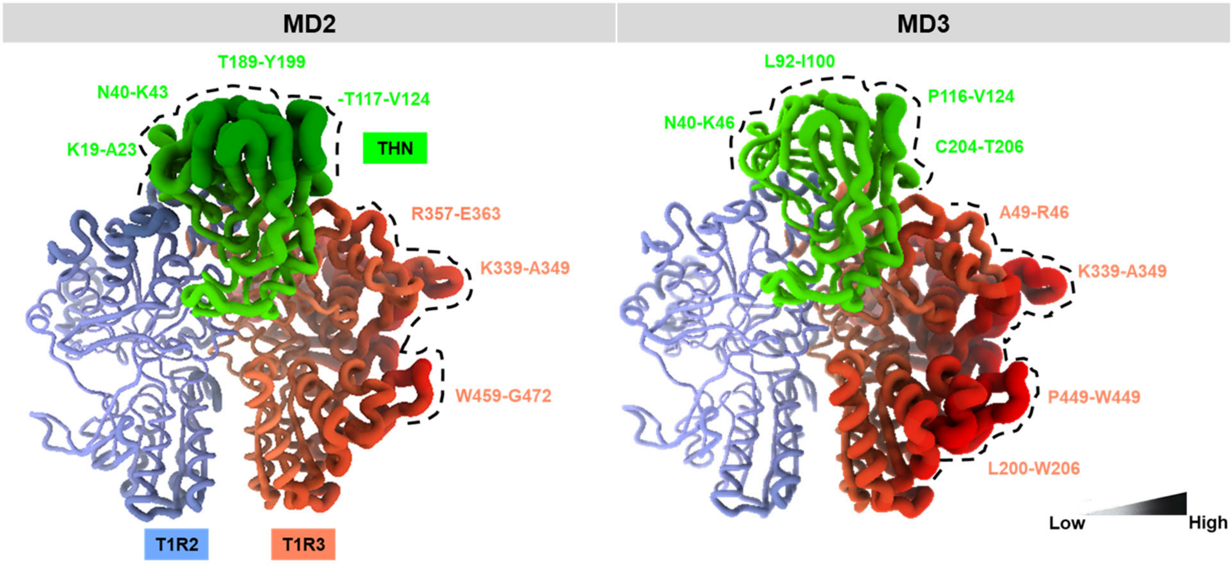

Figure S9. Representation of the protein-protein complex flexibility by B-factor in MD2-3. Local structure is ranked from rigid (lighter gradient, value of 0.0) to flexible (darker gradient, value of 70). The residues with high fluctuations corresponding to the high B-factor value have been highlighted. (L=Low; H=High).

Table S1. The interface statistics obtained from PDBsum server for T1R2/T1R3-thaumatin complexes.

	Chain	#interface residues	Interface area (Å^2)	#salt bridges	#H-bonds	#non-bonded contacts
**Open form**	T1R3: THN	10:9	558:554	-	1	27
**Closed form**	T1R2: THN	11:11	665:669	1	4	53
	T1R3: THN	3:3	188:194	1	2	10
**AlphaFold2 Multimer**	T1R3: THN	15:20	944:798	3	2	152
**AlphaFold3**	T1R3: THN	13:9	543:603	-	2	63

Table S2. The inter and intra chain interaction comparison among the three MD runs with residues as edges and interactions as nodes.

	**MD1**		**MD2**		**MD3**	
	**Intrachain**	**Interchain**	**Intrachain**	**Interchain**	**Intrachain**	**Interchain**
**H-bond**	731	28	791	28	719	26
**pi-pi stack**	30	3	37	3	28	7
**pi-cation**	5	0	4	1	6	0
**Ionic**	29	4	20	6	25	5
**Pi-H bond**	2	0	2	1	1	0
**vdW**	6500	336	6425	412	6450	420
